# Association between metabolic control and oral health in children with type 1 diabetes mellitus

**DOI:** 10.1186/s12903-022-02555-x

**Published:** 2022-11-16

**Authors:** Lulëjeta Ferizi, Venera Bimbashi, Jeta Kelmendi

**Affiliations:** 1 Department of Pediatric and Preventive Dentistry, Dental School, Faculty of Medicine, University of Prishtina and University Dentistry Clinical Center of Kosovo, Prishtina, Republic of Kosovo; 2 Department of Prosthodontics, Dental School, Faculty of Medicine, University of Prishtina and University Dentistry Clinical Center of Kosovo, Rrethi i Spitalit p.n. 10000, Prishtina, Republic of Kosovo; 3 Department of Orthodontics, Dental School, Faculty of Medicine, University of Prishtina, Kosovo and University Dentistry Clinical Center of Kosovo, Prishtina, Republic of Kosovo

**Keywords:** Children, Type 1 diabetes mellitus, Oral health, Metabolic control

## Abstract

**Background:**

Type 1 diabetes mellitus is the most common chronic disease and can be reflected in the oral cavity. The aim of this study was to analyze the impact of metabolic control on saliva, dental caries, dental plaque, gingival inflammation, and cariogenic bacteria in saliva.

**Methods:**

A case–control epidemiologic study was performed on children with type 1 diabetes (ages 10–15 years) separated into two groups: 34 children with good metabolic control of glycated hemoglobin (HbA1c < 7.5%) and 46 children with poor metabolic control of glycated hemoglobin (HbA1c > 7.5%). Oral status was assessed using the Decay, Missing, and Filled Teeth index for permanent teeth (DMFT), plaque index and gingival index. The stimulated salivary flow rate was measured, and the colonies of *Streptococcus mutans* and Lactobacillus in saliva were determined. The observed children answered questions related to their frequency of brushing habits, dental visits and parents’ education. Mann–Whitney U Test, Chi-Square test and Fisher’s exact test were used in the statistical analyses. The significance level was set at *p* < 0.05.

**Results:**

The children with diabetes with poor metabolic control had significantly higher values of the DMFT index, plaque index, and gingival index, more colonies, and a higher risk of *Streptococcus mutans* and Lactobacillus than the children with diabetes with good metabolic control (*p* < 0.001). The level of metabolic control of diabetes had no influence on salivary flow rates (*p* > 0.05). The majority of both groups with good and poor metabolic control brushed their teeth once per day, and they visited the dentist only when necessary (61.3%). However, the parents of children in both groups had medium to low levels of education, without any significant difference between groups (*p* > 0.05).

**Conclusion:**

The results indicate that children with diabetes have a lower level of oral hygiene and are potentially to dental caries and periodontal diseases, mainly when their metabolic disorder is uncontrolled.

## Background

Type 1 diabetes (T1D) is a chronic autoimmune disease caused by the destruction of insulin-producing beta cells in the pancreas [1, 2]. This illness is more common in children and adolescents, although it can also affect adults [1, 3]. This is characterized by insulin deficiency, and patients develop life-threatening hyperglycemia that is clinically manifested by trio of the symptoms associated with disease, weight loss, polyuria, and polydipsia [4]. Initially, patients with type 1 diabetes lose control of blood glucose, which often can result in acute ketoacidosis and later with secondary complications such as heart disease, blindness and kidney failure [5].

Studies show that type 1 diabetes progresses, partially due to a combination of genetic predisposition (patients with type 1 diabetes have high-risk human leukocyte antigen-HLA genes) and mainly because of unknown environmental factors [6, 7]. Knowing that patients with type 1 diabetes mellitus have insulin deficiency, there is an immediate need for exogenous insulin replacement. This treatment is required throughout life. Insulin is usually obtained through subcutaneous injections, but today with technological development, insulin pumps help patients with type 1 diabetes manage their insulin administration [1].

The incidence and prevalence of type 1 diabetes are increasing worldwide, and this situation is quite distressing. The results of a meta-analysis showed that the incidence of type 1 diabetes was 15 per 100,000 people, and the prevalence was 9.5% worldwide [8]. According to this study, the incidence of type 1 diabetes in Asia, Europe and America was higher than that in Africa, whereas a high prevalence of type 1 diabetes occurs in Europe and America. This increase in the incidence and prevalence of type 1 diabetes in European and American countries was also related to lifestyle changes. Henceforth, in underdeveloped countries, a lower incidence and prevalence were found, where there is still a lack of insulin therapy due to the high prices of these medications, which poses a major problem in the management of type 1 diabetes [8].

The oral cavity is one of the most affected organs as a result of diabetes. Many studies have revealed a multilayered link between type 1 diabetes and dental caries, as these children have limited intake of carbohydrates, which are cariogenic foods [9–11]. Saliva is the secret of the three large salivary glands and a number of small salivary glands, which play a role in maintaining a healthy oral cavity and regulate the balance between demineralization and remineralization, preventing the occurrence of dental caries [12]. However, children with type 1 diabetes have a lower stimulated parotid gland flow rate, which is followed by a low buffering capacity and increasing levels of pathogenic bacteria that cause dental caries, such as *Streptococcus mutans* and Lactobacillus [12, 13]. Consequently, poor metabolic control of diabetes increases the level of action of the aforementioned factors [14].

Periodontal disease has been reported as a more frequent oral complication of diabetic children and is also similarly known as the sixth major complication of diabetes [15]. As a result of diabetes, blood capillary vessels in tissues and organs are affected, so similar changes can be found in the tissues of the mouth, particularly in the periodontium [16–18]. Periodontal diseases arise in childhood for children with diabetes and are closely related to poor metabolic control measured by the percentage of glycated hemoglobin (HbA1c). Consequently, there is an increase in the clinical dental plaque indices accompanying inflammation and gingival bleeding [19, 20].

Therefore, a great importance for children with diabetes is placed on both regular teeth brushing and regular dental visits. Good oral hygiene prevents dental caries and periodontal disease and correspondingly affects the regulation of diabetic metabolic control [21].

Additionally, parental behavior greatly affects the health of their children. Thus, the level of education of the parents is extremely important in the precaution of the oral health of the children [12, 22].

However, there are insufficient studies on the correlation between oral health and metabolic control. Studies in both developed and developing countries have presented a significant impact that poor metabolic control has on the incidence of dental caries, reducing the salivary flow rate, increasing the level of cariogenic bacteria in saliva and causing inflammatory changes in periodontal tissue [14, 23–27]. Observance blood glucose levels under control develop more challenging as children grow older due to the impact of pubertal hormones and the failure of self-care practices.

Unfortunately, most patients with diabetes neglect oral health care and face problems related to diabetes and metabolic control. Therefore, the necessity of this study was an attempt to identify the state of oral health and to inform the parents of children with diabetes about the need for oral treatments as well as the needful preventive measures for this medically compromised patient group.

The aim of the study was to assess oral health among children with type 1 diabetes mellitus related to metabolic control of glycated hemoglobin (HbA1c).

## Methods

### Study design and protocol

A case–control epidemiologic study was performed among 80 children with type 1 diabetes (ages 10–15 years) who were receiving treatment for type 1 diabetes at the Pediatric Clinic at the University Clinical Center of Kosovo. Parents of children with diabetes were advised to send their children for a dental visit. The sample consisted of the random selection of children aged 10–15 years. From the 106 patients who were receiving treatment for type 1 diabetes at the Pediatric Clinic at the University Clinical Center of Kosovo, 80% were randomly selected, or 80 children. The sample size was calculated using Cohran formula, with a confidence level of 95% and a confidence interval of 2 [28]. To avoid the effect of confounders in the stages of designing and analyzing the study, we selected the specific age group of children with type 1 diabetes mellitus with good and poor metabolic control.

The data pertaining to glycated hemoglobin during the last 6 months were extracted from their medical records. According to the American Diabetes Association, good metabolic control in children was considered when the glycated hemoglobin (HbA1c) value was 7.5% or 58 mmol/mol [29]. Therefore, based on information on the state of their metabolic control in the last 6 months, children with diabetes mellitus type 1 were divided into two groups: children with type 1 diabetes mellitus with good metabolic control (HbA1c < 7.5%, *n* = 34) and children with type 1 diabetes mellitus with poor metabolic control of glycated hemoglobin (HbA1c > 7.5%, *n* = 46). In addition, the inclusion criteria were that there were no other systemic diseases in their health history and that they had not taken antibiotics within the previous month. Additionally, both groups of children who had a meal within an hour before examination were excluded from CRT analysis. The exclusion criteria for both groups were patients with systemic treatment with antibiotics in the last month and patients with other diseases but not related to diabetes who were under the treatment for that disease.

The study was performed in accordance with the Declaration of Helsinki, and ethical approval for this study was obtained from the Ethics Committee-Medical Faculty, University of Prishtina, Kosovo with Reference Number 4000/2016. The study was conducted during the period January 2017 to June 2018. Written informed consent was obtained from the parents of the children involved in this study.

### Clinical examination

Clinical oral examinations were performed in the Department of Pediatric and Preventive Dentistry, University Dentistry Clinical Center of Kosovo (UDCCK), by a pediatric dentist researcher. Patients were examined under an artificial light using a dental mirror and a dental probe. All examinations were carried out by LF, with intra-examiner reliability of kappa = 0.95 based on the examination of all children (they were examined two times by same examiner). To determine the reliability of dental plaque and gingival condition, the dental assistant was asked to arrange for one in 10 children to be re-examined by another researcher. This was done without the examiner’s knowledge (LF), and the examination and re-examination were separated by at least 1 h. Re-examination of approximately 10% of the children resulted in 80 children being evaluated by the different examiner (JK). For testing inter-examiner agreement, Kappa statistics were calculated (0.96).

The clinical dental health status using the decayed, missing and filled teeth (DMFT) index for permanent teeth was scored according to the examination protocol that has been advocated by the World Health Organization diagnostic criteria for epidemiological studies [30].

The children were examined while seated on the dental chair for the amount of dental plaque and gingival condition, which were assessed at 4 sites (mesiobuccal, distobuccal, mesiolingual and distolingual). Silness and Löe criteria were used to quantify plaque deposition (plaque index-PI), whereas gingival status was assessed using the gingival index (GI) of Löe and Silness [31, 32].

### Microbiological procedures

Subjects were given thorough instructions beforehand regarding the procedures pertaining to saliva collection. They were also told not to eat or drink at least 2 hours before sampling and were asked to refrain from oral hygiene procedures such as brushing with fluoridated toothpaste at least 1 h prior to salivary sample collection. Saliva was always collected 2 h after the last meal and at the same time of day (12:00 midday). This was done to standardize the procedure of saliva collection. Drinking water was given to the subjects to rinse their mouths. Five minutes after the oral rinse, stimulated saliva was collected. Initially, patients were given paraffin pellets to stimulate saliva for 5 min. During that time, the saliva was collected in a sterile container, and the stimulated salivary flow rate of each patient was measured. The presence of *Streptococcus mutans* (SM) and Lactobacillus (LB) was determined using the CRT bacteria test (Ivoclar Vivadent, Liechtenstein) on saliva previously stimulated by chewing paraffin. Bacterial counts were recorded as colony-forming units per milliliter (CFU/mL) of saliva. Following the manufacturers’ scoring card, the number of bacterial colonies for *Streptococcus mutans* was graded as follows: Class 0 (none detected), Class 1 (10^2^–10^3^ CFU/mL), Class 2 (10^4^–10^5^ CFU/mL), and Class 3 (CFU ≥ 10^5^/mL), while the number of Lactobacillus colonies was graded as follows: Class 1 (none detected), Class 2 (10^2^–10^3^ CFU/mL), Class 3 (10^4^-10^5^ CFU/mL), and Class 4 (CFU ≥ 10^5^/mL). Moreover, findings lower than 10^5^ CFU/mL indicate a low risk for caries (Classes 0 and 1 for SM/Classes 1 and 2 for LB), whereas findings higher than 10^5^ CFU/mL indicate a high risk for caries disease (Classes 2 and 3 for SM/Classes 3 and 4 for LB).

The bacterial CRT test reacts more selectively, thus allowing early detection of *Streptococcus mutans* and Lactobacillus. Additionally, the use of saliva instead of dental plaque increased the efficiency of representation of the available microflora in the oral cavity.

### Questionnaire

After the clinical examination and under the supervision of their respective parents, the children were given a questionnaire for completing. The questions were about gender and residence, frequency of brushing habits, frequency of dental visits, and parents’ education. Each question consisted of three options, such as brushing habits, which were as follows: twice a day, once a day and rarely. The frequency of dental visits was once every 6 months, once a year, and only when necessary. In addition, last, about parents’ education level, low-level (had attended only primary school); medium level (attended high school level education), and high-level of education (had achieved a university or college degree).

### Statistical analysis

Data analysis was performed using SPSS 19 (SPSS Inc., Chicago, Illinois, USA) and Excel 2010 (Microsoft Corporation, Redmond, WA, USA). The difference in the values of stimulated salivary flow rate, DMFT index for permanent teeth, plaque index and gingival index between groups related to metabolic control of glycated hemoglobin (HbA1c) was tested using the Mann–Whitney U Test. The differences in the series with attributive traits between the two groups (HbA1c < 7.5% and HbA1c > 7.5%) were tested using Pearson Chi-square, Fisher’s Exact Test, and Fisher’s Exact Test/Monte Carlo Sig./(p). The level of significance for all tests was set at *p* < 0.05.

## Results

This study was carried out on 80 subjects with type 1 diabetes mellitus, out of which 34 had good-controlled metabolic control (HbA1c < 7.5%) and 46 had poor metabolic control (HbA1c > 7.5%) of the disease. The gender and residence factors were not found to have a significant impact within the two metabolic control groups among children with diabetes (*p* > 0.05) (Table 1).

**Table 1:** The differences among gender and residence between groups related to metabolic control

	Groups				Total		Test
	HbA1c < 7.5%		HbA1c > 7.5%				
	N	%	N	%	N	%	
**Gender**							
Male	16	47.1	23	50.0	39	48.8	Chi = 0.07 *p* > 0.05
Female	18	52.9	23	50.0	41	51.3	
**Residence**							
Urban	18	52.9	20	43.5	38	47.5	Chi = 0.70 *p* > 0.05
Rural	16	47.1	26	56.5	42	52.5	
**Total**	34	100.0	46	100.0	80	100.0	

The differences between the two groups, pertaining to stimulated salivary flow rate, DMFT index, plaque index, and gingival index, are presented in Table 2, which indicates that subjects with current poor metabolic controls (HbA1c > 7.5%) had significantly more dental caries, dental plaque, and gingivitis than subjects with good metabolic control (HbA1c < 7.5%) (*p* < 0.001). However, the stimulated salivary flow rate did not show any significant differences between the two groups among children with diabetes (*p* > 0.05).

**Table 2:** Differences compared in stimulated salivary flow rate, DMFT index, plaque index, and gingival index between groups based on metabolic control

Variable	Rank sum of HbA1c < 7.5%	Rank sum of HbA1c > 7.5%	HbA1c < 7.5% (*N* = 34) Mean ± SD	HbA1c > 7.5% (*N* = 46) Mean ± SD	*p* value
Stimulated salivary flow rate	1562.50	1677.50	0.89 ± 0.16	0.83 ± 0.16	*p* > 0.05
DMFT index	970.50	2269.50	4.74 ± 1.80	7.91 ± 3.94	*p* < 0.001
Plaque index (PI)	797.50	2442.50	1.78 ± 0.36	2.23 ± 0.28	*p* < 0.001
Gingival index (GI)	889.00	2351.00	0.79 ± 0.58	1.46 ± 0.50	*p* < 0.001

Mann-Whitney U Test *p ≤ 0.05

Furthermore, the study results revealed that all children with diabetes had *Streptococcus mutans* (SM) class 0 deficiency. The SM prevalence in both groups of children was 23.5 and 2.2% (Class 1). Classes that represented a higher risk for caries (Classes 2 and 3) were present in 47.1 and 29.4% in the good-controlled diabetes group and 23.9 and 73.9% in the poorly controlled diabetic group, respectively. Correspondingly, there was a significant difference between both groups for colonies of SM (divided into classes) (*p* < 0.001). Additionally, there was a significant SM (*p* < 0.01) difference between groups pertaining to the risk of caries. From the findings, both groups were predisposed for having a high risk of caries (76.5 and 97.8%) (Table 3).

**Table 3:** General and specific distribution of *Streptococcus mutans* between groups

	Groups				Total		Fisher test
	HbA1c < 7.5%		HbA1c > 7.5%				
	N	%	N	%	N	%	
***Streptococcus mutans*** **(SM)**							
Class 1 (10^2^–10^3^ CFU/mL)	8	23.5	1	2.2	9	11.3	Fisher test = 18.10 *p* < 0.001
Class 2 (10^4^–10^5^ CFU/mL)	16	47.1	11	23.9	27	33.8	
Class 3 (≥10^5^ CFU/mL)	10	29.4	34	73.9	44	55.0	
**SM values in CFU/mL saliva (Caries risk test for SM)**							
Low [< 10^5^ (0 and 1)]	8	23.5	1	2.2	9	11.3	*p* < 0.01
High [≥ 10^5^ (2 and 3)]	26	76.5	45	97.8	71	88.8	
**Total**	34	100.0	46	100.0	80	100.0	

The distribution of Lactobacillus (LB) into classes among both groups of children with diabetes is shown in Table 4. Class 1 LB was not recorded among any of the children with diabetes. Children with diabetes with good metabolic control had significantly higher levels (29.4%) of colonies of LB in Class 2 in comparison to poor metabolic control (21.7%). Class 3 of LB tended to be similar in both groups of children with diabetes (29.4 and 21.7%). Nevertheless, regarding Class 4 of LB, poorly controlled children with diabetes had higher levels of colonies of LB (73.9%) than well-controlled children with diabetes (11.8%) (*p* < 0.001). Regarding the caries risk of LB, children with diabetes and poor metabolic control had a significantly higher risk for caries than diabetic children with good metabolic control (95.7 and 41.2%, respectively) (*p* < 0.001).

**Table 4:** General and specific distribution of Lactobacillus between groups

	Groups				Total		Chi test
	HbA1c < 7.5%		HbA1c > 7.5%				
	N	%	N	%	N	%	
**Lactobacillus (LB)**							
Class 2 (10^2^–10^3^ CFU/mL)	20	58.8	2	4.3	22	27.5	Chi = 37.45 *p* < 0.001
Class 3 (10^4^–10^5^ CFU/mL)	10	29.4	10	21.7	20	25.0	
Class 4 (≥10^5^ CFU/mL)	4	11.8	34	73.9	38	47.5	
**LB values in CFU/mL saliva (Caries risk test for LB)**							
Low [< 10^5^ (1 and 2)]	20	58.8	2	4.3	22	27.5	*p* < 0.001
High [≥ 10^5^ (3 and 4)]	14	41.2	44	95.7	58	72.5	
**Total**	34	100.0	46	100.0	80	100.0	

Regarding brushing habits and dental visits, the study found no significant difference between the groups (Table 5). The majority of children brushed their teeth only once per day (61.3%), and they visited the dentist only when necessary (61.3%) (*p* > 0.05). Similarly, there was no significant difference between groups pertaining to children’s parents’ education level (*p* > 0.05). Most of the parents among both groups had medium to low levels of education (Table 5).

**Table 5:** Brushing habits, dental visits and parent’s education between groups

	Groups				Total		Test
	HbA1c < 7.5%		HbA1c > 7.5%				
	N	%	N	%	N	%	
**Brushing habits (per day)**							
Once a day	18	52.9	31	67.4	49	61.3	Chi = 1.99 *p* > 0.05
Twice a day	10	29.4	8	17.4	18	22.5	
Rarely	6	17.6	7	15.2	13	16.3	
**Dental visits**							
Once in 6 months	4	11.8	8	17.4	12	15.0	Chi = 4.40 *p* > 0.05
Once a year	12	35.3	7	15.2	19	23.8	
Only when necessary	18	52.9	31	67.4	49	61.3	
**Father’s education**							
Low level	5	14.7	14	30.4	19	23.8	Fisher = 4.77 *p* > 0.05
Medium level	25	73.5	31	67.4	56	70.0	
High level	4	11.8	1	2.2	5	6.3	
**Mother’s education**							
Low level	16	47.1	30	65.2	46	57.5	Fisher = 2.96 *p* > 0.05
Medium level	16	47.1	15	32.6	31	38.8	
High level	2	5.9	1	2.2	3	3.8	

## Discussion

Diabetes mellitus is a common chronic metabolic disease with numerous oral and systemic manifestations [33]. Children with diabetes have many problems during their lifetime, and dental and oral health problems are among those. Oral manifestations of diabetes include dental caries, salivary dysfunction, oral mucosa and other oral infections, taste and neurosensory disorders, gingivitis, periodontitis, etc. [12, 33–35]. Previously conducted studies in Kosovo compared the oral health of children with diabetes and control groups [12, 36, 37], whereas our study goes deeper and reveals the impact of metabolic control on oral health among children with diabetes. Many studies have shown that one of the most evident oral symptoms of diabetes is a reduction in the salivary flow rate. At the same time, this is supplemented by an increase in glucose levels not only in the blood but also in saliva [38–40]. The same data indicate a low level of saliva flow related to children with diabetes, as reported in other studies [14, 41, 42]. According to our study, the stimulated salivary flow rate was similar in both groups, which is congruent with the results of the research performed by other studies [24, 43]. However, xerostomia not only reduces the amount of saliva but also negatively affects the quality of life of children with diabetes [44].

Many studies conducted on dental caries in Kosovo, both in healthy children and those with diabetes, have shown a higher value of dental caries [12, 36, 37, 45–48] in children with diabetes. This study compares the dental caries situation of children with diabetes, although with different metabolic control levels of the disease. The results of this study reveal that the DMFT score is higher in children with poorly controlled diabetes. Higher values of dental caries among children with poor control of diabetes were observed, studied, and presented by other authors [12, 23, 26, 36, 37, 49]. Moreover, Orbak et al., in their study, found that the subjects with poor metabolic control, in addition to slightly more caries, also had a higher incidence of caries in permanent dentition [26]. Furthermore, the association between poor metabolic control and dental caries was unveiled by other studies [23, 24, 50, 51]. Conversely, there are other studies that did not find any significant relationship between caries level and metabolic control of diabetes [43]. Nevertheless, the relatively high percentage of diabetes-caused oral complications, in particular dental caries, are associated with poor metabolic control of diabetes. However, the reason for higher DMFT in diabetic subjects in our study is not only uncontrolled diabetes but also poor oral hygiene. In this paper’s study, the diabetic subjects with poor metabolic control exhibited a higher plaque index (PI) and gingival index (GI) than the subjects with good metabolic control diabetes, which is consistent with the results of other findings [52]. However, there are published data that have reported no significant differences between plaque index and gingival index related to diabetic children with metabolic control [24, 50]. However, when compared to the control group, most studies in children with diabetes found high values of plaque index and gingival index [26, 36]. Diabetes increases the risk of gingivitis and periodontitis. Periodontal disease is considered one of the complications of diabetes, and this connection has been recognized in the dental literature for many decades [53]. According to studies, periodontal disease in children with diabetes appears to progress around puberty because puberty adversely impacts insulin action and HbA1c control [52–54]. Studies have shown that the prevalence, severity, and progression of periodontal diseases are significantly increased in patients with diabetes, especially where diabetes is associated with poor metabolic control [53]. Previously, it was shown that periodontal disease affects glycemic control and that this connection is bilateral; with worsening glycemic control, periodontal disease is exacerbated and vice versa [26, 53–56].

Dental caries is a disease that involves multiple factors that coincide at a certain point and at a certain time. The basic factors are the presence of the causal microorganism (*Streptococcus mutans* is the main cause of initial caries, while Lactobacillus is more related to a later stage of caries development), the host (tooth), the substrate (diet) and the patient’s immune capacity [27, 30]. Our study findings reveal higher values of *Streptococcus mutans* and Lactobacillus colonies and a higher risk of dental caries in children with uncontrolled diabetes. This is consistent with other clinical studies that reported a correlation between the metabolic control of diabetic patients and the concentrations of saliva *Streptococcus mutans* and Lactobacillus [23, 24, 57]. Nevertheless, unlike the aforementioned studies, other authors reported no association with bacterial colonies and metabolic control of diabetes [27, 43]. However, most of the studies show a distinct association between higher levels of *Streptococcus mutans* and Lactobacillus and the presence of dental caries in patients with diabetes with poor metabolic control [23, 25, 51]. High levels of these bacteria in saliva are an indicator of a cariogenic environment in the mouths of diabetic patients, particularly uncontrolled diabetes subjects. Therefore, it can be concluded that poor glycemic control promotes the growth of *Streptococcus mutans* and Lactobacillus in the saliva of type 1 diabetes mellitus patients. Likewise, salivary factors play important roles in controlling the salivary status of cariogenic bacteria [57, 58].

Brushing habits and dental visits are considered to be the main methods to prevent oral diseases, including gingivitis and dental caries. These study results indicated that most diabetic children brush their teeth only once per day and visit the dentist only when necessary. The same results regarding the frequency of tooth brushing were found in other studies [31, 50, 59], whereas few studies have reported better results regarding brushing habits, where a higher percentage of children with diabetes brush their teeth two to three times a day [21, 60].

Studies related to children with type 1 diabetes mellitus rarely visit dentists, which is congruent with our findings [12, 23]. However, other study findings have stated that children with type 1 diabetes visit a dentist regularly [20, 59, 61]. When considering dental health assessment, it is interesting to note that children with diabetes do not assess their oral health. They are focused on systemic disease, and they have no knowledge of the risks posed by poor oral health or the impact of oral health on the metabolic control of diabetes.

On a slightly different note, Moore et al. stated that one of the reasons that subjects with diabetes avoided dental visits was the high cost of dental care [62].

A particular importance in children’s oral health is also the level of parents’ education. In our study, the level of parents’ education in both groups of children with diabetes was medium to low. Several studies reported similar findings regarding the education of parents of children with diabetes [12, 60], although there were also other study findings resulting in no relationship between metabolic control and parents’ education [56]. The low and medium levels of education of the parents could influence the children’s knowledge pertaining to the reciprocal connection between type 1 diabetes and oral health. Therefore, parents’ education may have a key role in the oral health of children with type 1 diabetes and their metabolic control of the disease.

Our study has several strengths. To our knowledge, this is the first study in Kosovo that evaluates the relationship between metabolic control and oral health in children with type 1 diabetes mellitus. Furthermore, we simultaneously evaluated various oral health components in relation to metabolic control of disease in the same group of children. Finally, this study will be a strong basis for further studies in our country regarding the impact of metabolic control of diabetes on oral health.

Additionally, age group is limited and there was 5 year gap in age of participation, and no information has been given as to whether age had any influence in the outcome between the youngest or oldest, and primary teeth are not included, hence the authors suggest further studies. However, the results of this study provide an overview of oral health among children with type 1 diabetes and its relationship to metabolic control of the disease.

## Conclusion

The findings of this study suggest that children with poor diabetic control tend to have a reduction in salivary flow rate, a high risk of caries, plaque accumulation, gingival inflammation, and an increase in bacterial colonies. Hence, children with diabetes are advised to brush their teeth and visit the dentist regularly. Furthermore, it is recommended that parents obtain better knowledge and are aware of the impact of metabolic control on the oral health of diabetic children.

## Data Availability

The data used to support the findings of this study are available from the corresponding author upon reasonable request.

## Abbreviations

HbA1c: Glycated hemoglobin
DMFT: Decayed, missing due to caries, and filled teeth in the permanent teeth
T1DM: Type 1 diabetes mellitus
PI: Plaque index
GI: Gingival index
SM: *Streptococcus mutans*
LB: Lactobacillus
